# Trajectories of affective disorders—the central structures of CRC/TRR 393

**DOI:** 10.1007/s00115-025-01921-8

**Published:** 2025-11-26

**Authors:** Udo Dannlowski, Andrea Pfennig, Ulrich W. Ebner-Priemer, Frederike Stein, Ralph Müller-Pfefferkorn, Michael N. Smolka, Andreas Jansen, Tim Hahn, Hamidreza Jamalabadi, Benjamin Straube, Irina Falkenberg, Tilo Kircher, Nina Alexander

**Affiliations:** 1 Institut für Translationale Psychiatrie, Medizinische Fakultät, Universität Münster, Albert-Schweitzer-Campus 1, Gebäude A9a, 48149 Münster, Germany; 2 Universitätsklinikum Carl Gustav Carus, Klinik und Poliklinik für Psychiatrie und Psychotherapie, Technische Universität Dresden, Dresden, Germany; 3 Fakultät für Geistes- und Sozialwissenschaften, Institut für Sport und Sportwissenschaft, Karlsruher Institut für Technologie, Hertzstraße 16, 76187 Karlsruhe, Germany; 4 Department of Psychiatry and Psychotherapy, Central Institute of Mental Health, Medical Faculty Mannheim, University of Heidelberg, Mannheim, Germany; 5 Fachbereich Medizin, UKGM, Universitätsklinik für Psychiatrie und Psychotherapie, Universität Marburg, Marburg, Germany; 6 Zentrum für Informationsdienste und Hochleistungsrechnen (ZIH), Technische Universität Dresden, Dresden, Germany; 7 Department of Psychiatry, Medical School and University Medical Center OWL, Protestant Hospital of the Bethel Foundation, Bielefeld University, Bielefeld, Germany

**Keywords:** Mood disorders, Major depressive disorder, Bipolar disorder, Course of illness, Clinical depression, Stimmungsschwankungen, Major Depression, Bipolare Störung, Krankheitsverlauf, Klinische Depression

## Abstract

The recurrent and often unpredictable course of affective disorders poses a critical challenge for long-term patient care. The CRC/TRR 393 consortium has established an ambitious longitudinal study, the German Mental Health Cohort (GEMCO), to systematically investigate the trajectories of symptom recurrence and remission in affective disorders. This article provides an overview of the core structural projects of the CRC/TRR 393 consortium that underpin this effort. *Project S02* orchestrates the GEMCO, recruiting 1500 participants (approximately 900 with major depressive disorder, 300 with bipolar disorder, 300 healthy controls) and conducting comprehensive phenotyping, neuroimaging, and biobanking at baseline and follow-up time points. *Project S01* provides an innovative mobile health infrastructure for continuous monitoring of patients’ mood, behavior, and environment in real time over a 2-year period, enabling detection of early warning signs (“inflection signals”) of mood episodes. *Project INF* implements a centralized information infrastructure, ensuring high-quality data capture, multisite data integration, and open-science data sharing. *Project S03* serves as the advanced data analysis hub, developing machine learning models to predict individual illness trajectories and outcomes from the rich multimodal data. A research training group (RTG) provides funding and infrastructure for early-career scientists. Together, these structural projects establish a state-of-the-art framework for studying affective disorder trajectories, with the ultimate goal of identifying predictors and mechanisms of relapse and remission, and paving the way toward mechanism-based clinical interventions.

## Abstract (de)

Der Verlauf affektiver Störungen stellt eine zentrale Herausforderung für die langfristige Versorgung dar. Der SFB/TRR 393 hat mit der German Mental Health Cohort (GEMCO) eine ambitionierte Langzeitstudie etabliert, um systematisch Verläufe affektiver Störungen zu untersuchen. Dieser Beitrag gibt einen Überblick über die zentralen Strukturprojekte des Konsortiums. *Projekt S02* koordiniert die GEMCO und rekrutiert 1500 Teilnehmende – etwa 900 mit Major Depression (MDD), 300 mit bipolarer Störung (BD) und 300 gesunde Kontrollpersonen (HC). Es erfolgen umfassende Phänotypisierung, Bildgebung und Biobanking sowohl zu Studienbeginn als auch bei Follow-up-Erhebungen. *Projekt S01* stellt eine innovative mobile Gesundheitsinfrastruktur bereit, um Stimmung, Verhalten und Umweltfaktoren der Patient*innen über einen Zeitraum von 2 Jahren in Echtzeit zu erfassen. Damit können frühe Warnzeichen („inflection signals“) für Krankheitsepisoden erkannt werden. *Projekt INF* entwickelt eine zentrale Informationsinfrastruktur, die eine qualitativ hochwertige Datenerhebung, die standortübergreifende Integration der Daten sowie deren offene wissenschaftliche Nutzung ermöglicht. *Projekt S03* fungiert als Analysezentrum für die komplexen multivariaten Daten und entwickelt maschinelle Lernverfahren (ML), um individuelle Krankheitsverläufe und -ausgänge vorherzusagen. Ein integriertes Graduiertenkolleg („research training group“, *RTG*) stellt darüber hinaus Förderung und Infrastruktur für Nachwuchswissenschaftler bereit. Gemeinsam schaffen diese Strukturprojekte ein Rahmenwerk zur Untersuchung der Verlaufsmuster affektiver Störungen mit dem Ziel, prädiktive Marker und zugrunde liegende Mechanismen von Rückfällen und Remissionen zu identifizieren – und damit den Weg für klinische Interventionen zu ebnen.

## Introduction

Affective disorders, including major depressive disorder (MDD) and bipolar disorder (BD), are highly prevalent, often chronic conditions marked by recurrent episodes of depression and (in BD) mania or hypomania. Despite therapeutic advances, many patients experience relapses, and their timing and triggers remain difficult to predict. A substantial proportion of patients with MDD have multiple episodes, while patients with BD typically cycle through mood episodes throughout life. Identifying factors that distinguish stable remission from impending relapse is thus a major research priority. This requires mapping illness trajectories over time and detecting dynamic changes in symptoms and underlying biology that precede, accompany, or follow relapse or recovery. By clarifying these patterns—especially during early “inflection signals”—CRC/TRR 393 aims to enable earlier intervention and more personalized care [3]. A CRC (Collaborative Research Center) is a highly competitive grant by the Deutsche Forschungsgemeinschaft (DFG), which is funding the implementation of the present initiative.

To meet these challenges, the consortium’s overarching goal is to prospectively study patients over time to identify predictors and modulators of mood episode recurrence and remission, integrating perspectives from clinical psychology, psychiatry, neuroscience, and data science. Central to this effort is the establishment of a large, deeply phenotyped longitudinal cohort: the German Mental Health Cohort (GEMCO), combining patients with affective disorders and healthy controls. GEMCO builds on two major studies—Marburg–Münster Affective Disorders Cohort Study (DFG-FOR 2107) and Early-BipoLife—by re-recruiting participants for extended follow-up. Leveraging existing baseline data from over 4100 individuals, GEMCO provides an exceptionally rich dataset. Approximately 1500 participants are enrolled and assessed over at least 2 years at multiple time points.

A longitudinal study of this scale and complexity necessitates a robust and well-integrated research infrastructure. Accordingly, CRC/TRR 393 includes several structural projects whose role is to provide the essential backbone for data collection, data management, and data analysis across the consortium. These projects ensure standardized protocols, high data quality, cutting-edge methodology, and structured support for early-career scientists. In this review, we summarize five key structural projects—S02, S01, INF, S03, and RTG—highlighting their objectives, methods, and interconnections in supporting GEMCO and the overarching aims of CRC/TRR 393.

## S02: the GEMCO cohort—recruitment, phenotyping, neuroimaging, and biobanking

*Project S02* is the central hub of CRC/TRR 393, responsible for establishing and maintaining GEMCO. Its primary mission is to recruit and longitudinally follow up a well-characterized cohort, conducting comprehensive assessments at defined time points. In essence, S02 provides the human cohort infrastructure for the entire consortium. The cohort comprises approximately 1500 individuals, including patients with affective disorders (both MDD and BD) as well as healthy control (HC) participants. Importantly, these participants are predominantly drawn from two existing longitudinal studies: the *DFG Research Unit 2107* cohort (also known as the Marburg–Münster Affective Disorders Cohort Study, MACS; [4]), and the *Early-BipoLife* study cohort. The FOR 2107 cohort enrolled thousands of individuals (over 2800, including patients with affective disorder and controls), and the Early-BipoLife study similarly followed up about 1200–1400 at-risk patients and patients with early BD [6]. By contacting participants from these studies and inviting them into GEMCO, S02 leverages extensive baseline data already collected (on clinical history, demographics, etc.) and adds new prospective follow-up data. Participants are stratified by age to ensure a broad coverage of the lifespan from 16 to 65. Key inclusion criteria are the absence of contraindications to magnetic resonance imaging (MRI) and, for patients, a diagnosis of MDD or BD. Individuals with severe medical illnesses or active substance use disorders are excluded to reduce confounding influences. Both currently ill and remitted patients are included, as well as individuals at elevated risk for mood disorders (e.g., those with prodromal bipolar symptoms), in addition to healthy controls. This broad sampling framework allows the consortium to study not only active episodes but also transitions into episodes (depression or mania) and recoveries, across a spectrum from health to illness.

### Assessment and follow-up.

Once enrolled in GEMCO, all participants undergo a core battery of assessments at *three fixed time points*: baseline (T1), 12-month follow-up (T2), and 24-month follow-up (T3). At each of these time points, thorough *phenotyping* of participants is conducted. This includes diagnostic evaluations and symptom ratings (using structured clinical interviews such as the Diagnostic Interview for Psychiatric Disorders [DIPS] and standardized questionnaires for depression, mania, anxiety, etc.), cognitive testing, and measures of psychosocial functioning and life events. The total assessment time is ~ 100 min of interviewer-led assessment, ~ 130 min of questionnaires, and ~ 50 min of neuropsychological tests (see Table 1).

**Table 1:** Common battery of clinical rating scales and neuropsychological tests administered to the CRC/TRR 393 cohort at T1, T2, and T3.

*General data/demographics*	
General quality of life	SF-12 [8]
Family history	CRC/TRR393 documentation
Employment/salary	CRC/TRR393 documentation
Smoking	–
Alcohol consumption	Audit [9]
Drugs	CRC/TRR393 documentation
Nutrition	FFQ2 (short) [10]
Past and current medication	CRC/TRR393 documentation
Handedness	EHI [11]
History of somatic disorders	CRC/TRR393 documentation
Religiosity	CRC/TRR393 documentation
*Psychopathology*	
Diagnosis	DSM‑V [12], DIPS [13]
Clinical course	Life chart [14]
Depression level	BDI-II [15], HAM‑D [16]
Suicidality	SBQ‑R [17]
Mania symptoms	YMRS [18], ASRM [19]
Anxiety	HAM‑A [20], STAI-S/T [21]
Positive symptoms	SAPS [22]
Negative symptoms	SANS [23]
Anhedonia	SAS/PAS [24]
Formal thought disorder	TALD [25]
Global/social functioning	GAF [26], SOFAS [27], FAST [28], WHODAS 2.0 [29]
Bipolar disorder at-risk screening	BPSS-AS‑P [30]
Lithium response	Alda [31]
Optimism/pessimism	SOP [32]
Attachment style	RSQ [33]
Personality	NEO-FFI [34]
Self-efficacy	ASKU/GSE‑3 [36]
*Risk factors*	
Maltreatment	CTQ [37], ACE [38]
Life events	LEQ [39]
Perceived stress	PSS [40]
Loneliness	UCLA-Loneliness [41]
*Protective factors*	
Resilience	CD-RISC [42], BRCS [43]
Social network	SNI [44]
Social support	F‑SozU [45]
Parental bonding	FEB [46]
*CRC/TRR 393 core constructs*	
Interpersonal reactivity	IRI (SPF) [35]
Emotion regulation	CERQ [47], DERS [48], NMR-SF [49]
Cognitive–behavioral rhythms	MTCQ [50], ISI [51]
Expectation	G‑EEE [52]
*Neuropsychology*	
Verbal working memory	Letter–number span [53]
Verbal episodic memory	VLMT [54]
Verbal intelligence	MWT‑B [55]
Verbal fluency	RWT [56]
Executive functioning	TMT [57], symbol coding [58]
Attention	d2 [59]
Visuospatial working memory	Spatial span [60]

*ACE *Adverse Childhood Experiences, *ASKU/GSE-3 *Allgemeine Selbstwirksamkeit Kurzskala/General Self-Efficacy Short Scale 3,* ASRM *Altman Self-Rating Mania Scale, *BDI *Beck’s Depression Inventory, *BPSS-AS‑P* Bipolar Prodrome Symptom Scale–Abbreviated Screen for Patients, *BRCS* Brief Resilience Coping Scale, *CD-RISC* Connor–Davidson Resilience Scale, *CERQ* Cognitive Emotion Regulation Questionnaire, *CTQ* Child Trauma Questionnaire, *DERS* Difficulties in Emotion Regulation Scale, *DIPS *Diagnostic Interview for Psychiatric Disorders,* DSM-V *Diagnostic and Statistical Manual of Mental Disorders Fifth Edition, *EHI *Edinburgh Handedness Inventory, *FAST* Functional Assessment Screening Tool,* FFQ2* Food Frequency Questionnaire 2, *F‑SozU* Fragebogen zu Sozialer Unterstützung, *GAF* Global Assessment of Functioning, *G‑EEE* Generic rating scale for previous treatment experiences, treatment expectations, and treatment effects, *HAM‑A* Hamilton Rating Scale for Anxiety, *HAM‑D* Hamilton Rating Scale for Depression, *IRI (SPF)* Interpersonal Reactivity Index (Saarbrücken Personality Questionnaire), *ISI* Insomnia Severity Index, *LEQ* Life Events Questionnaire, *NEO-FFI* NEO Five-Factor Inventory, *MTCQ* Munich Chronotype Questionnaire, *MWT‑B* Mehrfachwahl-Wortschatz-Intelligenztest, *NMR-SF* Negative Mood Regulation Scale – Short Form, *PSS* Perceived Stress Scale, *RSQ* Relationship Scales Questionnaire, *RWT* Regensburger Wortflüssigkeits-Test, *SAS/PAS* Chapman Social/Physical Anhedonia Scale, *SBQ‑R* Suicide Behaviors Questionnaire–Revised, *SANS* Scale for the Assessment of Negative Symptoms, *SAPS* Scale for the Assessment of Positive Symptoms, *SF-12* 12-Item Short-Form Health Survey, *SNI* Social Network Index, *SOFAS* Social and Occupational Functioning Assessment Scale, *SOP* Optimism–Pessimism Short Scale, *STAI-S/T* State–Trait Anxiety Inventory, *TALD* Thought and Language Disorder Scale, *TMT* Trail Making Test, *VLMT* Verbal Learning and Memory Test, *WHODAS 2.0* World Health Organization Disability Assessment Schedule 2.0, *YMRS* Young Mania Rating Scale

**Fig. 1:**
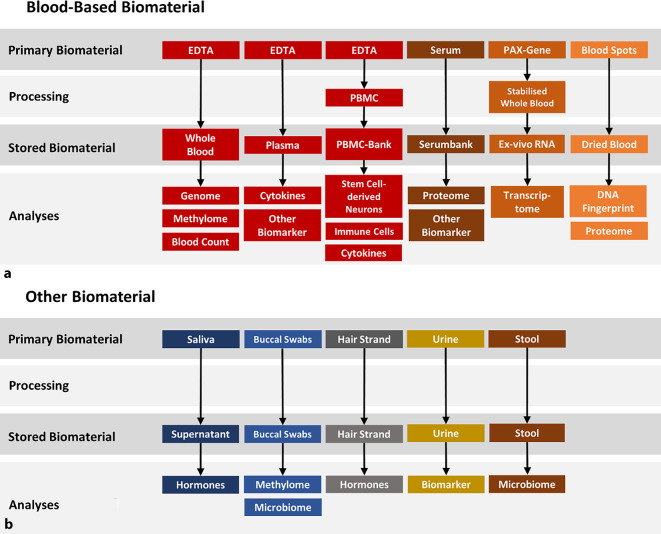
**a, b** Biobanking workflow in the CRC/TRR 393 GEMCO cohort

In addition to clinical and behavioral data collection, S02 coordinates biological sampling (biobanking) and neuroimaging procedures within the cohort. At each assessment point, participants provide biological samples—including blood, buccal cells, saliva, hair, urine, and stool—which are processed and stored for downstream analyses of genetic, epigenetic, immune, hormonal, and other biomarkers (see Fig. 1). These biosamples will enable later studies of biological predictors or correlates of mood disorder trajectories (for instance, identifying inflammatory markers associated with relapse).

**Fig. 2:**
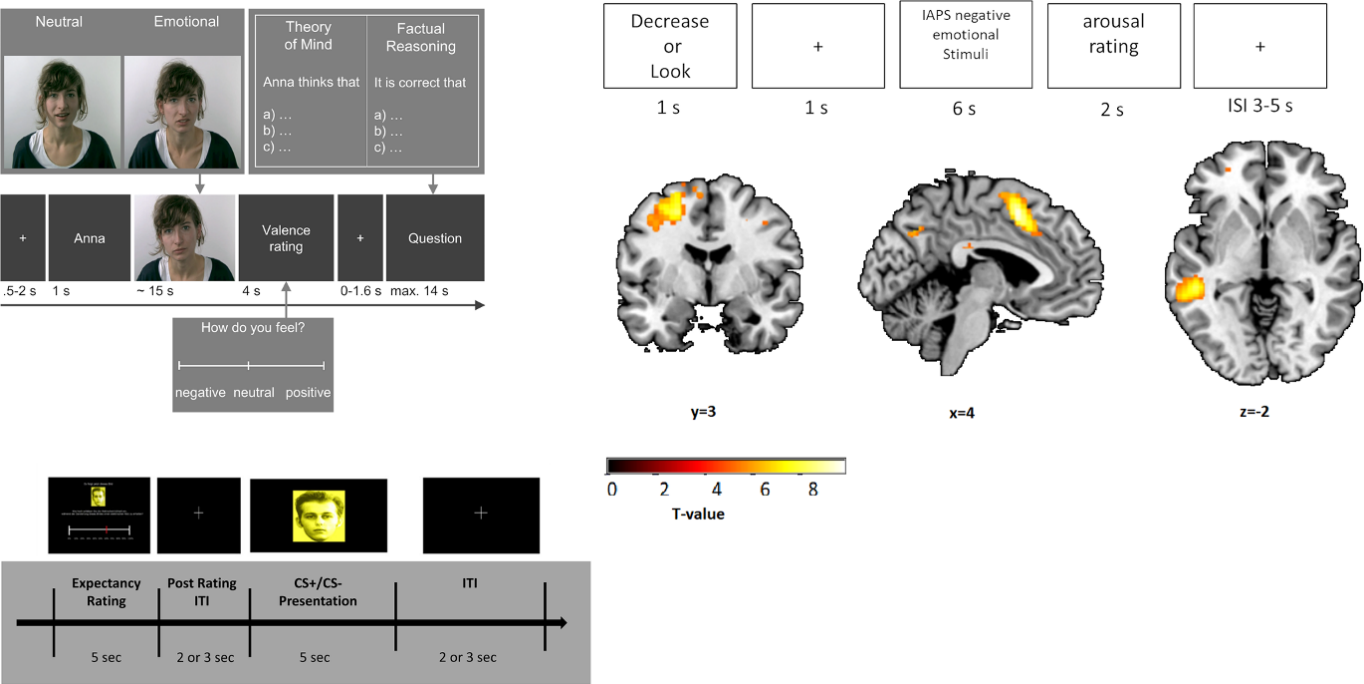
Three functional magnetic resonance imaging paradigms employed in the imaging battery. Top left, the EmpaToM paradigm eliciting social cognition and theory of mind processes [61]. Top right, a classic emotion regulation paradigm with stimuli from the IAPS-database [62] elicits neural processing of emotional stimuli. Bottom, the expectation paradigm reflects a conditioning task eliciting expectation processes to aversive stimuli [63]. (Adapted from [63])

Likewise, participants undergo standardized *MRI* and *electroencephalogram* (*EEG)* protocols at baseline and follow-ups, including structural MRI and functional MRI sequences. High-resolution T1-weighted structural MRI and diffusion tensor imaging (DTI) enable quantification of brain anatomy and white matter connectivity, while functional MRI (e.g., resting-state or task-based paradigms) provides measures of brain activity (see Fig. 2). Repeated MRI assessments at 1‑ and 2‑year intervals allow S02 to establish a longitudinal neuroimaging dataset to examine brain changes over time in relation to clinical course. Harmonized standard operating procedures (SOPs) for MRI acquisition are implemented at all three sites to ensure data comparability, and quality assurance procedures (phantom scans, centralized quality checks) are in place as part of the INF project (see below) to maintain high data quality.

All clinical, cognitive, behavioral, and neuroimaging data collected in S02 are transferred to centralized databases in collaboration with Project INF. Joint quality control procedures ensure data completeness, consistency, and standardized preprocessing (e.g., artifact detection in MRI, aliquoting of biosamples).

Project S02 also coordinates participant recruitment and longitudinal tracking across sites, primarily drawing from prior cohorts. Recruitment teams manage scheduling, communication, and retention throughout the 2‑year follow-up.

As the central data-generating unit, S02 provides the participant pool and core data infrastructure for hypothesis-driven projects within CRC/TRR 393. Domain A projects [1] use S02 longitudinal data to investigate illness trajectories, while intervention studies in Domain C [5] recruit directly from the cohort. Coordination between S02 and individual projects ensures efficient use of resources and prevents participant overload, thereby facilitating data integration and synergy across the consortium.

In summary, Project S02 establishes GEMCO as a longitudinal resource for studying the course of affective disorders. By enrolling a large, well-characterized sample and conducting repeated deep phenotyping, S02 provides core data on illness trajectories, from clinical symptoms to brain changes. These data underpin the consortium’s efforts to understand relapse and recovery mechanisms. With follow-up planned beyond the initial 2 years (potentially up to 10–12 years), GEMCO will gain further value for studying long-term outcomes. S02 works closely with other structural projects, especially S01, which captures continuous data between major assessments.

## S01: mobile infrastructure for continuous and real-time monitoring

While S02 covers assessments at fixed intervals, Project S01 enables continuous monitoring between these time points. S01 implements a mobile assessment infrastructure that continuously tracks participants’ mood, behavior, and context in their daily lives over the entire 2‑year study period.

In effect, S01 extends the “follow-up” beyond the clinic visits, using digital health technology to capture symptom dynamics in real time. By continuous intensive sampling of data in daily life, S01 enables the detection of early changes and short-term fluctuations that might precede a full relapse or indicate impending improvement. These early changes have been termed “inflection signals,” reflecting symptom change that might foreshadow a major change in clinical state.

### Ambulatory assessment and mobile sensing.

S01 deploys smartphone-based monitoring to gather active self-reports, including ecological momentary assessments (EMA), and passive sensor measurements. A dedicated smartphone app will prompt participants every evening (end-of-day diary) to report on current mood, sleep quality, or other symptoms. This sampling yields a fine-grained longitudinal record of each individual’s affective state over the 2‑year period. In parallel, the smartphone collects passive data without requiring action from the participant: for instance, accelerometer data (physical activity), Global Positioning System (GPS) location (mobility patterns), phone usage metrics (e.g., frequency of calls or messages), and possibly wearable sensor readings (such as heart rate via a fitness band). These passive measures can serve as objective proxies for behavior and physiology (e.g., reduced mobility and activity might indicate a developing depressive phase) and impose minimal patient burden.

### Real-time detection of symptom changes.

**Fig. 3:**
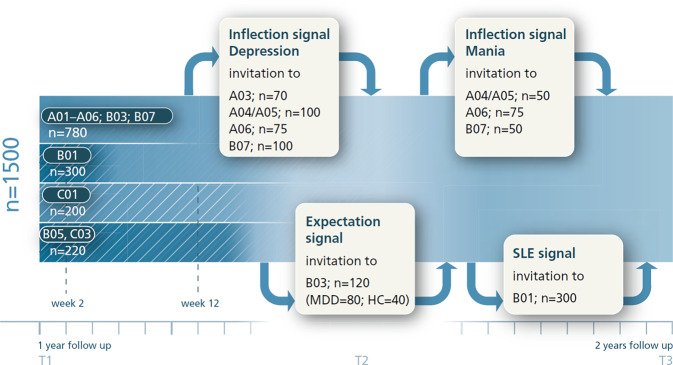
Patient flow from the mHealth infrastructure perspective. During CRC/TRR 393 recruitment, patients will be allocated to different mobile assessment project streams (inflection signal for mania/depression; emotion regulation intervention in C01; social interaction intervention in B05/C03). See [2, 5] for details of these projects. *SLE* stressful life events

Beyond collecting data, S01 is designed to perform real-time analysis of incoming data to identify significant changes. The project implements algorithms that monitor individuals’ mood ratings and sensor metrics for patterns suggestive of deterioration or improvement in mood symptoms. For instance, a sustained drop in self-reported mood or a sharp increase in sleep disturbances might indicate a potential depressive relapse. Similarly, signs of rising energy and reduced need for sleep could presage a hypomanic switch in bipolar disorder. Inflection signals (see Fig. 3) are detected in real time, prompting local teams to follow up with the corresponding patients.

Another innovative aspect is the linking of real-time monitoring with triggered interventions or assessments in other projects from Domains A, B, and C ([1, 2, 5]; see Fig. 3). The infrastructure of S01 actively coordinates with research projects that require timely engagement during specific mood states or events. For example, participants are asked to undergo additional examinations (such as an additional MRI or EEG) when they experience a significant mood change. S01 makes this feasible by promptly detecting those changes and alerting the relevant study team. In the consortium’s design, detection of an inflection signal can lead to “on-demand” activation of intensive data collection protocols in certain projects. As a concrete case, if a participant’s smartphone data indicate a likely relapse into depression, Projects A03–A06 [1] can be notified to invite that participant for specialized scans or blood tests at the earliest opportunity. Similarly, S01 monitors stressful life events (SLEs), such as significant personal stressors, which are hypothesized to trigger symptom changes. Upon detecting an SLE (via self-report or contextual data), S01 can initiate intensive e‑diary sampling and trigger additional questionnaires on social support or emotion regulation in projects investigating these domains.

Technologically, S01 builds upon proven platforms for mobile data collection. The project uses established software frameworks (such as the *movisensXS* system for Android devices and the InteractionDesigner) to implement the EMA schedules and sensor data integration. These frameworks have been used in prior large studies (including CRC/TRR 265 on addiction and BipoLife) and have demonstrated excellent usability and compliance even in long-term assessments. Data from the smartphones are transmitted securely (with end-to-end encryption) to central servers in real time, maintaining pseudonymization. S01 incorporates a compliance dashboard, tracking participants’ adherence to the protocol and can send reminders or alerts to study staff if a participant has not responded for a certain period. This ensures data completeness and timely support if participants disengage (e.g., via a support call).

## Project INF: information infrastructure and data management

**Fig. 4:**
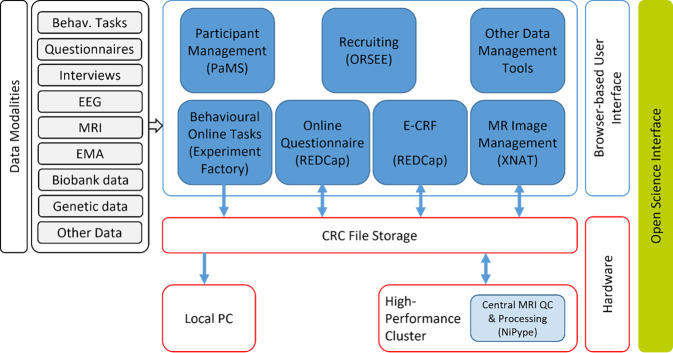
Architecture sketch of existing and planned CRC services in the INF project. *EEG* electroencephalogram, *EMA* ecological momentary assessments, *MRI* magnetic resonance imaging

The breadth and volume of data generated by S02 and S01—along with other projects in CRC/TRR 393—require a robust strategy for data management. *Project INF* (Information Infrastructure) handles all aspects of data storage, organization, and sharing within the consortium (and beyond). Its overarching goal is to ensure that the data collected are FAIR (findable, accessible, interoperable, and reusable; [7]), in line with modern Open Science principles. INF provides the technical environment, software tools, and protocols for integrating the diverse data streams (clinical, behavioral, neuroimaging, biomarker, etc.) from multiple sites and modalities into a coherent, high-quality database (Fig. 4).

One of the primary tasks of INF is to establish a central research data platform that all projects will use for entering and accessing data, including electronic case report forms (eCRFs) for clinical and questionnaire data, directly linking the assessments in S02 to a central server. As participants complete interviews or tests, the data are entered into and immediately synchronized with the central database. Similarly, MRI data are transferred to a centralized repository, and biosamples are catalogued in a central biobank information system. INF is also responsible for migrating existing data from earlier cohorts into this new infrastructure as well, including FOR 2107 and BipoLife. In essence, INF creates a comprehensive data warehouse for GEMCO, bringing together past and present data across sites.

The INF project implements automated quality control (QC) pipelines for both clinical and neuroimaging data. On the clinical side, this might involve range checks (e.g., ensuring that questionnaire scores fall within possible limits), logical checks (e.g., flagging a form if it is partially incomplete), and tracking missing data. Feedback to the sites is generated if data entries seem erroneous. For neuroimaging, INF coordinates standardized QC and preprocessing pipelines for all MRI data.

Another key aspect is ensuring standardized metadata. INF establishes common documentation for the data—e.g., standardized codes for diagnoses, or common labels for variables—so that everyone in the consortium “speaks the same language” when referring to data. Researchers querying the database will have clear documentation of each variable (with codebooks, data dictionaries) and can combine data from different modalities correctly.

Given the sensitive nature of personal health data, INF prioritizes data security and governance. All data are stored on secure servers with controlled access—researchers can only access de-identified data relevant to their project and only after appropriate approvals. Personal identifying information is kept separate and protected, likely in a trusted third-party or coded manner, to comply with data protection regulations (GDPR).

At the same time, INF facilitates *data sharing and accessibility* for legitimate research use. Within the consortium, it provides tools such as a web-based data portal where authorized investigators can query the cohort data, generate summary statistics, or extract an anonymized dataset for analysis. Over the course of the project, INF will also guide the process of making datasets available to the broader scientific community (following FAIR principles and journal/data policy requirements). This might include creating a curated database or contributing to public repositories. INF ensures that the valuable data collected can have an impact beyond just the immediate TRR projects, fostering replication and new discoveries by external scientists.

In conclusion, Project INF is the *digital and organizational nexus* of the CRC/TRR 393 research data. By establishing a centralized, high-quality data environment, INF enables the large-scale integrative analyses that the consortium aims for. It addresses a central challenge in modern psychiatric research: managing big data that are multimodal, longitudinal, and multicenter—including questionnaires, MRI, mobile phone logs, and genetic information. This integration is essential for the next project, S03, which will perform sophisticated analyses on the combined data.

## S03: machine learning and statistical modeling hub

Large, complex datasets like those produced by S02, S01, and INF offer an unprecedented opportunity to discover patterns that might predict individual outcomes—but they also pose significant analytical challenges. *Project S03* is the dedicated *analysis and modeling core* of the CRC/TRR 393 consortium, tasked with developing and applying advanced statistical and machine learning (ML) methods to the consortium’s data. In effect, S03 serves as an internal analytical consultancy and innovation unit: It provides expertise in handling high-dimensional data, creates predictive models for clinical trajectories, and ensures that cutting-edge techniques (such as deep learning, multivariate pattern analysis, and integrative modeling) are accessible to all TRR 393 projects.

### Consortium-wide data analysis.

S03 builds predictive models of disease course using the full breadth of data from the entire GEMCO cohort (*n* = 1500). This involves aggregating data types—clinical histories, symptom time series from S01, neuroimaging measures from S02, cognitive test results, biomarkers—and using ML algorithms to find combinations of features that forecast key outcomes. A primary prediction target is the occurrence and number of relapsing episodes. For example, S03 aims to predict upcoming episodes over the 2‑year follow-up. Another target is predicting the frequency of inflection signals. By training algorithms on the rich baseline and longitudinal data, S03 seeks to identify individuals at highest risk for relapse versus those on more stable trajectories, using digital phenotypes (patterns from smartphone EMA and sensing via S01), clinical/questionnaire data (psychiatric assessments and patient-reported outcomes), imaging-derived phenotypes (brain structure or connectivity metrics from MRI), and intensive sampling data (e.g., specific EMA bursts around events) as predictors. S03 then employs a range of ML techniques to build predictive models. These include classic ML models such as *random forests* and *kernel-based methods* as well as more complex *deep learning* approaches aiming to use pre-trained deep neural networks (e.g., architectures initially trained on large datasets) and fine-tune them on TRR 393 data to detect subtle patterns that simpler methods might miss. In addition, S03 will integrate these ML methods with dynamical systems modeling to enable the identification and eventually control of symptom and—where relevant—neural dynamics.

### Support for individual projects.

Beyond the big-picture cohort modeling, S03 actively collaborates with multiple research projects in Domains A, B, and C to address their data-analytic needs. Many projects in CRC/TRR 393 collect specialized datasets—for instance, high-density EEG recordings, behavioral task data, or even rodent behavioral data—and plan to use sophisticated analyses. S03 provides consulting and hands-on assistance to these projects.

From a scientific standpoint, S03 will explore novel methodological questions that arise from the consortium’s data. One example is handling temporal and multimodal data: Participants have both slow-varying data (e.g., MRI at yearly intervals) and fast-varying data (daily mood from EMA). S03 may investigate advanced statistical models that can incorporate both—such as hierarchical or dynamical models that link short-term fluctuations to long-term outcomes. Another example is the interpretability of ML models. S03 will apply techniques to interpret the model, potentially identifying new risk factors. Through such efforts, the work of S03 will not only yield predictive tools but also generate hypotheses about mechanisms, feeding back into the scientific understanding of affective disorders.

### Research Training Group (RTG)

**Fig. 5:**
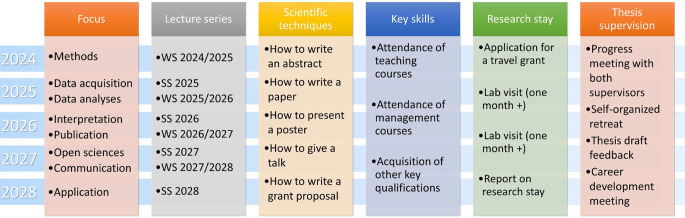
Qualification concept and curriculum of the integrated research training group

Project RTG (research training group) provides a training and exchange platform for CRC/TRR 393 early-career scientists. Interdisciplinary research within CRC/TRR 393 is essential, as expertise across multiple disciplines is required for completing a doctoral degree (PhD or MD). The integrated RTG will provide a platform for the structured training of doctoral candidates. It will build on the excellent existing university structures and successfully operating RTGs/IRTGs of the three partner sites and provide a sophisticated training program specific to the CRC/TRR 393 topics. This will enable our doctoral candidates to master their thesis projects with excellence on an internationally competitive level. Key elements of the RTG will be (a) lectures covering all domains of CRC/TRR 393; (b) workshops and (c) training sessions covering scientific techniques and key skills regarding lab management; (d) specific lab visits for (international) networking and knowledge acquisition; and (e) structured, continuous supervision, which will closely accompany each candidate throughout the development of their thesis. All this will be complemented by student retreats and journal clubs, method workshops, and soft skill training camps, which will be provided in collaboration with established local early-career structures. RTG will train a new generation of interdisciplinary and collaborative researchers with knowledge in all areas relevant for Domains A, B, and C of the proposed CRC/TRR 393 (see Fig. 5).

## Conclusion

In conclusion, the structural projects S02, S01, INF, and S03 constitute an integrated and methodologically aligned framework that enables the CRC/TRR 393 “Trajectories of Affective Disorders” consortium to generate and exploit one of the most comprehensive longitudinal datasets worldwide on depression and bipolar disorder—not only to predict individual illness trajectories, but to uncover the mechanisms that drive them.

## Declarations

For this article no studies with human participants or animals were performed by any of the authors. All studies mentioned were in accordance with the ethical standards indicated in each case.
